# Determinants of susceptibility to SARS-CoV-2 infection in murine ACE2

**DOI:** 10.1128/jvi.00543-25

**Published:** 2025-05-12

**Authors:** Takashi Kondo, Rigel Suzuki, Hisano Yajima, Sachiho Kawahara, Kodai Yamaya, Takaya Ichikawa, Shuhei Tsujino, Saori Suzuki, Tomokazu Tamura, Takao Hashiguchi, Takasuke Fukuhara

**Affiliations:** 1 Department of Microbiology and Immunology, Faculty of Medicine, Hokkaido University 12810, Sapporo, Japan; 2 Institute for Vaccine Research and Development: IVReD, Hokkaido University 12810, Sapporo, Japan; 3 Laboratory of Medical Virology, Institute for Life and Medical Sciences, Kyoto University 12918, Kyoto, Japan; 4 Department of Hematology, Faculty of Medicine, Hokkaido University 592506, Sapporo, Japan; 5 One Health Research Center, Hokkaido University 12810, Sapporo, Japan; 6 Kyoto University Immunomonitoring Center, Kyoto University 12918, Kyoto, Japan; 7 Laboratory of Virus Control, Research Institute for Microbial Diseases, Osaka University 34822 , Suita, Japan; 8 Department of Virology, Faculty of Medical Sciences, Kyushu University 12923, Fukuoka, Japan; 9 AMED-CREST, Japan Agency for Medical Research and Development (AMED) 444274, Tokyo, Japan; The Ohio State University, Columbus, Ohio, USA

**Keywords:** SARS-CoV-2, ACE2, spike protein, mice, rodent, variants, host tropism

## Abstract

Severe acute respiratory syndrome coronavirus 2 (SARS-CoV-2) utilizes angiotensin-converting enzyme 2 (ACE2) as a receptor to enter host cells, and primary receptor recognition of the spike protein is a major determinant of the host range of SARS-CoV-2. Since the emergence of SARS-CoV-2, a considerable number of variants have emerged. However, the determinants of host tropism of SARS-CoV-2 remain elusive. We conducted infection assays with chimeric recombinant SARS-CoV-2 carrying the spike protein from 10 viral variants, assessing their entry efficiency using mammalian ACE2 orthologs from species that have close contact with humans. We found that only murine ACE2 exhibited different susceptibilities to infection with the SARS-CoV-2 variants. Moreover, we revealed that the mutation N501Y in the viral spike protein has a crucial role in determining the infectivity of cells expressing murine ACE2 and of mice *in vivo*. Next, we identified six amino acid substitutions at 24, 30, 31, 82, 83, and 353 in murine ACE2 that allowed for viral entry of the variants to which murine ACE2 was previously resistant. Furthermore, we showed that ACE2 from a species closely related to mice, *Mus caroli*, is capable of supporting entry of the viral variants that could not use murine ACE2. These results suggest that few ACE2 orthologs have different susceptibility to infection with SARS-CoV-2 variants as observed for murine ACE2. Collectively, our study reveals critical amino acids in ACE2 and the SARS-CoV-2 spike protein that are involved in the host tropism of SARS-CoV-2, shedding light on interspecies susceptibility to infection.

**IMPORTANCE:**

SARS-CoV-2 can infect many species besides humans, leading to the evolution of the virus and adaptation to other animal hosts, which could trigger a new COVID-19 wave. The SARS-CoV-2 spike protein utilizes ACE2 as a receptor for entry into host cells. The interaction of ACE2 with the spike protein determines the host range of SARS-CoV-2. In this study, using chimeric viruses carrying the spike protein of SARS-CoV-2 variants to infect cells expressing different ACE2 orthologs from species humans come in close contact with, we confirmed murine ACE2 alone showed different susceptibility to the variants. We identified residues in murine ACE2 and the viral spike that restrict viral entry. Furthermore, an ACE2 ortholog from a species genetically close to mice mediated entry of SARS-CoV-2 variants incapable of infecting mice. This research highlights the uniquely limited susceptibility of mice to different SARS-CoV-2 variants and provides invaluable insights into the host tropism of SARS-CoV-2.

## INTRODUCTION

Severe acute respiratory syndrome coronavirus 2 (SARS-CoV-2) was first discovered in late 2019 in Wuhan, China (1). This highly contagious virus is the causative agent of coronavirus disease 2019 (COVID-19) and has led to millions of infections and deaths.

SARS-CoV-2 utilizes angiotensin-converting enzyme 2 (ACE2) as a receptor to enter host cells (1). Upon binding to ACE2, the SARS-CoV-2 spike protein is cleaved, facilitating membrane fusion for viral entry (2, 3). The cleavage is mediated by either transmembrane protease serine 2 (TMPRSS2) on the cell surface or by cathepsin L in the endosomal compartment following ACE2-mediated endocytosis. Therefore, the interaction between the spike protein and ACE2 is a key determinant of SARS-CoV-2 host tropism.

As the pandemic continues, numerous SARS-CoV-2 variants have emerged (4). Based on their epidemiological and biological significance, the World Health Organization has classified these as variants of concern (VOCs) and variants of interest (VOIs). One of the main differences between SARS-CoV-2 VOCs/VOIs is the amino acid mutations in the spike protein, which can potentially alter host tropism (5, 6). ACE2 is widely expressed in various tissues and animal species, and different ACE2 orthologs can mediate SARS-CoV-2 entry, suggesting that the virus has a wide host range (7–10). Indeed, SARS-CoV-2 has been reported to infect not only humans but also other mammals. Viral transmission among animals can lead to the emergence of new variants, increasing the risk of human reinfection and triggering new COVID-19 outbreaks. Recent studies have identified white-tailed deer and mink as potential reservoirs of SARS-CoV-2 (11–13), and another study reported that imported hamsters contributed to the onset of a new COVID wave (14). In addition, other studies have suggested that the Omicron variant may have originated in mice (15–17). Therefore, it is crucial to understand the range of animal species susceptible to SARS-CoV-2 to prevent future pandemics. Rodents, in particular, require close monitoring, as they have been linked to the new wave of infection. While previous studies have investigated the susceptibility of mice and other animals to SARS-CoV-2 VOCs/VOIs (18–27), the precise determinants of SARS-CoV-2 host tropism remain unclear.

In this study, we assessed the ability of spike protein from 10 SARS-CoV-2 variants to infect cells expressing ACE2 orthologs from nine animals that have close contact with humans using live viruses. We observed that cells expressing murine ACE2 had varying susceptibility to the SARS-CoV-2 variants, whereas cells expressing other ACE2 orthologs supported entry of all 10 SARS-CoV-2 variants. We also found that the mutation N501Y in the spike protein has a crucial role in various SARS-CoV-2 variants to infect mice both *in vitro* and *in vivo*. Among ACE2 orthologs, we identified six key amino acid substitutions (N24Q, N30D, N31K, S82M, F83Y, and H353K) that allowed murine ACE2 to support the entry of SARS-CoV-2 variants that typically do not infect mice. Notably, we revealed that there are no animal species that have the same amino acids as murine ACE2 at the six sites mentioned above. Our analysis revealed that ACE2 from a rodent species closely related to mice, which shares five of these six residues, was able to support viral entry of variants that do not infect standard laboratory mice. These results suggest that mice are the only species exhibiting different susceptibility among SARS-CoV-2 variants, even when compared to closely related rodent species. Collectively, we identified the amino acid residues in murine ACE2 and mutations in the spike protein of SARS-CoV-2 that influence SARS-CoV-2 host susceptibility, highlighting the broad host range. Our findings provide new insights into the species tropism of SARS-CoV-2 infection, with implications for pandemic surveillance and risk assessment.

## RESULTS

### Murine ACE2 has differing susceptibility to SARS-CoV-2 variants

**Fig 1:**
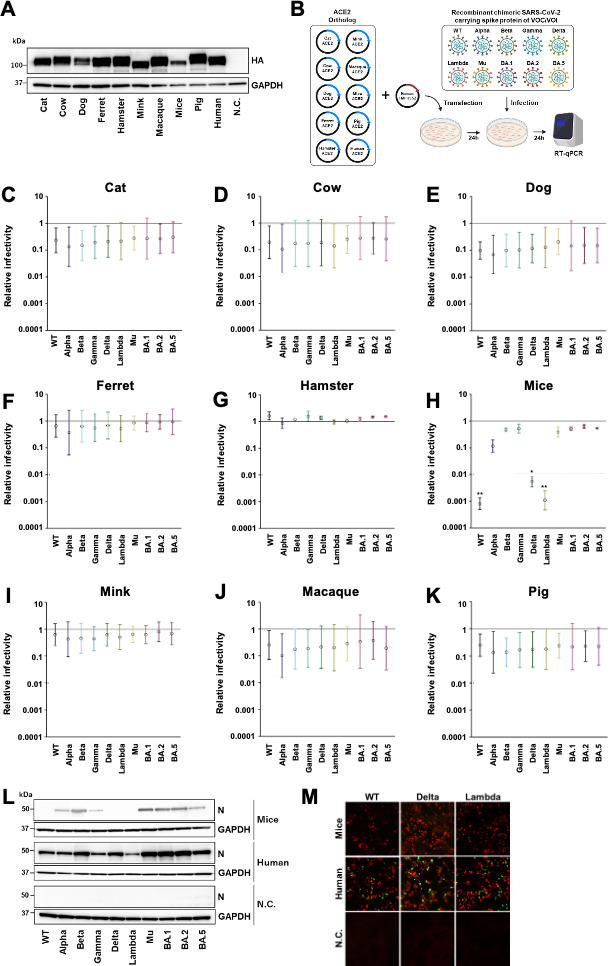
Infectivity of SARS-CoV-2 VOCs/VOIs in cell lines expressing murine ACE2. (**A**) The expression of the HA-tagged ACE2 orthologs in HEK293T cells was confirmed by western blot using an anti-HA antibody. GAPDH was used as a load control. N.C.: negative control. (**B**) An illustration showing the experimental workflow. ACE2 orthologs and human TMPRSS2 were transfected into HEK293T cells. After 24 h, cells were inoculated with chimeric recombinant SARS-CoV-2 VOC/VOIs (MOI = 0.1) and incubated for 24 h. Then cells were lysed, and the level of intracellular viral RNA was quantified by RT-qPCR. (**C through K**) Intracellular viral RNA levels following inoculation with the SARS-CoV-2 VOC/VOIs are presented as relative infectivity, with the levels observed in human ACE2-expressing cells set as 1. Data are shown for HEK293T cells expressing human TMPRSS2 and the ACE2 ortholog of cat (**C**), cow (**D**), dog (**E**), ferret (**F**), hamster (**G**), mouse (**H**), mink (**I**), macaque (**J**), or pig (**K**). Experiments were done in triplicate. ^*^P < 0.05 and ^**^P < 0.01. (**L and M**) Infection of cells expressing murine or human ACE2 was evaluated by western blot of viral nucleocapsid protein (**L**) and immunofluorescence of GFP from the recombinant chimeric SARS-CoV-2 (M; HA-tagged animal ACE2 in red and GFP signal in green).

As a first step in assessing the species tropism of SARS-CoV-2 variants, we used the circular polymerase extension reaction (CPER) method (28) to generate chimeric recombinant viruses based on the sequence of B.1.1 (WT), each encoding the spike protein from one of 10 VOCs/VOIs: B.1.1 (WT), B.1.1.7 (Alpha), B.1.351 (Beta), P.1 (Gamma), B.1.617.2 (Delta), C.37 (Lambda), B.1.621 (Mu), and three Omicron subvariants, BA.1, BA.2, and BA.5. Open reading frame 7a in each of these viruses was substituted with green fluorescent protein (GFP) to allow for monitoring of viral kinetics. In parallel, we constructed expression plasmids encoding ACE2 from nine mammals that have frequent contact with humans: *Felis catus* (cat), *Bos taurus* (cow), *Canis familiaris* (dog), *Mustela furo* (ferret), *Mesocricetus auratus* (hamster), *Neovison vison* (mink), *Macaca fascicularis* (macaque), *Mus musculus* (mice), and *Sus domesticus* (pig). The C-terminus of each ACE2 ortholog was fused with an HA tag, and expression was confirmed by western blotting (Fig. 1A).

HEK293T cells were transfected with these ACE2 plasmids along with a plasmid encoding human TMPRSS2, which facilitates SARS-CoV-2 infection. Twenty-four hours post-transfection, cells were infected with the chimeric recombinant SARS-CoV-2 spike protein variants, and viral RNA was quantified at 24 h post-infection (h.p.i.; Fig. 1B). To evaluate the extent to which each ACE2 ortholog could support uptake of the SARS-CoV-2 variants, the amount of intracellular viral RNA was calculated and expressed relative to the amount observed in cells expressing human ACE2, which was set as 1. We found that cells expressing the ACE2 ortholog from cat (Fig. 1C), cow (Fig. 1D), dog (Fig. 1E), ferret (Fig. 1F), hamster (Fig. 1G), mink (Fig. 1I), macaque (Fig. 1J), and pig (Fig. 1K) were susceptible to all 10 SARS-CoV-2 variants.

In contrast, cells expressing murine ACE2 were susceptible to the Alpha, Beta, Gamma, Mu, BA.1, BA.2, and BA.5 variants but not WT, Delta, or Lambda (Fig. 1H). We solubilized the cells expressing murine ACE2 24 h.p.i. to assess the presence of SARS-CoV-2 nucleocapsid by western blot. Nucleocapsid protein was only detected in cells that had been infected with the SARS-CoV-2 variants that also led to the presence of intracellular viral RNA (Alpha, Beta, Gamma, Mu, BA.1, BA.2, and BA.5 but not WT, Delta, and Lambda; Fig. 1L). To further confirm these findings, we used immunofluorescence microscopy with anti-HA tag and anti-GFP antibodies to detect exogenous ACE2 and SARS-CoV-2, respectively. No GFP signal was detected in the murine ACE2-expressing cells infected with WT, Delta, or Lambda (Fig. 1M). These results substantiated that murine ACE2-expressing cells are not susceptible to infection with the WT, Delta, and Lambda spike protein variants. Moreover, of the nine orthologs we investigated, only murine ACE2 showed differing susceptibility to the SARS-CoV-2 variants.

### The spike protein mutation N501Y enables SARS-CoV-2 utilization of murine ACE2 *in vitro*

**Fig 2:**
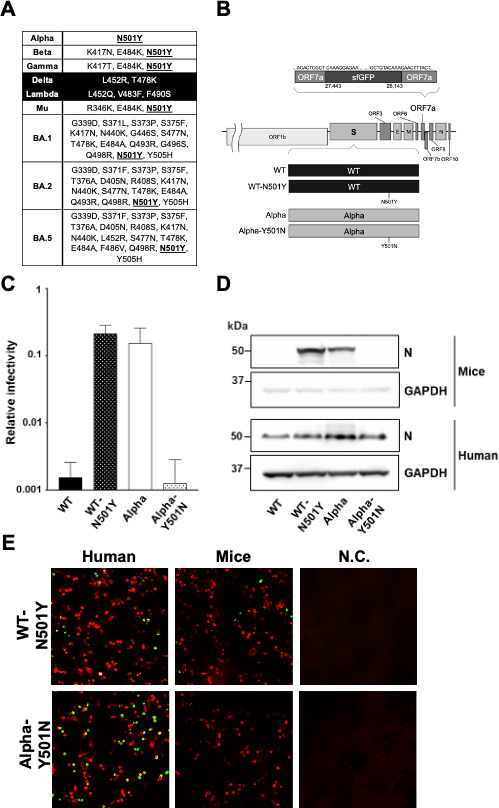
The susceptibility of cells expressing murine ACE2 to SARS-CoV-2 with the spike mutation N501Y. (**A**) Amino acid mutations in the RBD of 10 SARS-CoV-2 variants compared with WT. The variants which do not infect mice are shaded in black. The mutation N501Y was commonly found across SARS-CoV-2 variants that infect mice and is underlined. (**B**) Scheme for the chimeric recombinant viruses with the N501Y mutation in the spike protein of WT (WT-N501Y) or the Y501N mutation in the spike protein of Alpha (Alpha-Y501N). (**C**) The relative infectivity of SARS-CoV-2 carrying the spike protein of WT, WT-N501Y, Alpha, or Alpha-Y501N in cells expressing murine ACE2 vs human ACE2. Intracellular viral RNA levels following inoculation with the chimeric recombinant SARS-CoV-2 (MOI = 0.01) are presented as relative infectivity, with the levels observed in human ACE2-expressing cells set as 1. (**D and E**) Murine ACE2-expressing HEK293T cells were infected with SARS-CoV-2 carrying the WT, WT-N501Y, Alpha, or Alpha-Y501N spike protein. Infection of cells expressing murine or human ACE2 was evaluated by western blot of viral nucleocapsid protein (**D**) and immunofluorescence of GFP from the recombinant chimeric SARS-CoV-2 (E; HA-tagged animal ACE2 in red and GFP signal in green).

To investigate the amino acid residues within the SARS-CoV-2 spike protein that may be involved in determining the ability of murine ACE2 to support viral entry, we aligned the receptor-binding domain (RBD) sequences of the spike proteins of the 10 SARS-CoV-2 variants we were using (Fig. 2A). We found that N501Y was a common mutation in the SARS-CoV-2 variants we identified as infecting murine ACE2-expressing cells. To understand the effect of this mutation further, we introduced it into the WT strain (WT-N501Y) and conversely reversed the mutation to the Alpha strain (Alpha-Y501N; Fig. 2B). Subsequently, we used these viruses to infect cells expressing human or murine ACE2 and quantified intracellular viral RNA by RT-qPCR (Fig. 2C). As expected, viral RNA levels were greater in WT-N501Y-infected vs WT-infected cells expressing murine ACE2, while the reverse mutation in Alpha-Y501N decreased its infectivity compared to the parental Alpha strain. Furthermore, viral nucleocapsid was only detected in the murine ACE2-expressing cells infected with WT-N501Y and Alpha and not in those infected with WT and Alpha-Y501N (Fig. 2D). Similarly, GFP signal, a proxy for viral infection, was only detected by immunofluorescence in murine ACE2-expressing cells infected with WT-N501Y but not with Alpha-Y501N (Fig. 2E). These results suggest that the N501Y mutation is important in determining SARS-CoV-2 entry via murine ACE2.

### The spike protein mutation N501Y enables SARS-CoV-2 utilization of murine ACE2 *in vivo*

**Fig 3:**
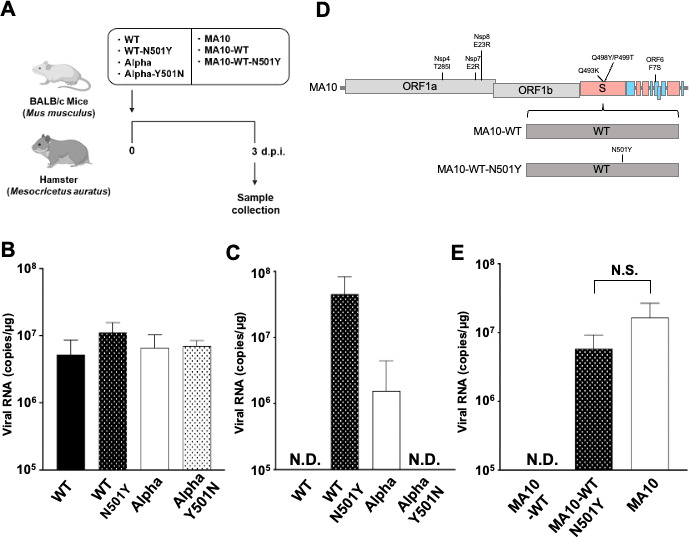
Infection with mutant viruses *in vivo*. (**A**) Illustration of the experimental workflow. Syrian hamsters (4-week-old, male) and BALB/c mice (8-week-old, male) were intranasally inoculated at 5,000 TCID_50_ with recombinant chimeric SARS-CoV-2 WT (*n* = 4), WT-N501Y (*n* = 4), Alpha (*n* = 4), Alpha-Y501N (*n* = 4), MA10 (*n* = 4), MA10-WT (*n* = 4), or MA10-WT-N501Y (*n* = 4). At 3 d.p.i., mice and hamsters were euthanized and lung tissue collected. (**B**) Viral RNA loads in the lung hilum of hamsters infected with WT, WT-N501Y, Alpha, or Alpha-Y501N at 3 d.p.i. were quantified by RT-qPCR. (**C**) Viral RNA loads in the lung hilum of BALB/c mice infected with WT, WT-N501Y, Alpha, or Alpha-Y501N at 3 d.p.i. were quantified by RT-qPCR. One mouse inoculated with WT virus unexpectedly died during the experiment. (**D**) Schema of the chimeric recombinant viruses SARS-CoV-2 MA10, MA10-WT (the spike protein of MA10 replaced by that of WT), and MA10-WT-N501Y (where MA10 spike was replaced with that of WT carrying the N501Y mutation). (**E**) Viral RNA loads in the lung hilum of mice infected with MA10, MA10-WT, or MA10-WT-N501Y were quantified by RT-qPCR. N.D.: not detected. N.S.: no significance.

We then assessed the infectivity of WT, WT-N501Y, Alpha, and Alpha-Y501N *in vivo* in hamsters, a standard animal model for SARS-CoV-2 infection (29, 30). Four-week-old Syrian hamsters (*n* = 4) were inoculated intranasally, and the amount of viral RNA in the lungs was quantified 3 days post-infection (3 d.p.i.; Fig. 3A). The viral RNA loads in the lung hilum were similar across the four viruses evaluated (Fig. 3B), indicating that in hamsters, these viruses have comparable infectivity.

Next, to investigate whether the effects of the N501Y mutation on utilization of murine ACE2 *in vitro* were also applicable *in vivo*, we performed infection experiments using mice. Eight-week-old BALB/c mice (*n* = 4) were inoculated intranasally, and the amount of viral RNA was quantified at 3 d.p.i. (Fig. 3A). Viral RNA loads in the lung hilum of mice infected with WT-N501Y or Alpha were higher than those of mice infected with WT or Alpha-Y501N (Fig. 3C), mirroring our *in vitro* findings.

To further investigate the importance of the N501Y mutation in mice, we performed infections with mouse-adapted SARS-CoV-2 MA10 (31). MA10 has seven mutations (Nsp4: T285I; Nsp7: E2R; Nsp8: E23R; S: Q493K, Q498Y, and P499T; ORF6: F7S) to facilitate murine infection and have N at 501 position (Fig. 3D). In this study, we generated MA10-based chimeric viruses in which the MA10 spike protein was replaced with either WT spike (MA10-WT) or WT spike with the N501Y mutation (MA10-WT-N501Y). We observed that the viral RNA loads in the lung hilum of mice were higher following infection with MA10-WT-N501Y or MA10 vs MA10-WT. Moreover, viral RNA loads did not significantly differ between mice infected with MA10-WT-N501Y or MA10 (Fig. 3E). This indicates that the N501Y mutation enhances the susceptibility of mice to SARS-CoV-2 in a manner comparable to what is observed for the mouse-adapted virus MA10.

### The amino acid residues at positions 24, 30, 31, 82, 83, and 353 in murine ACE2 are critical determinants of SARS-CoV-2 infectivity in mice

**Fig 4:**
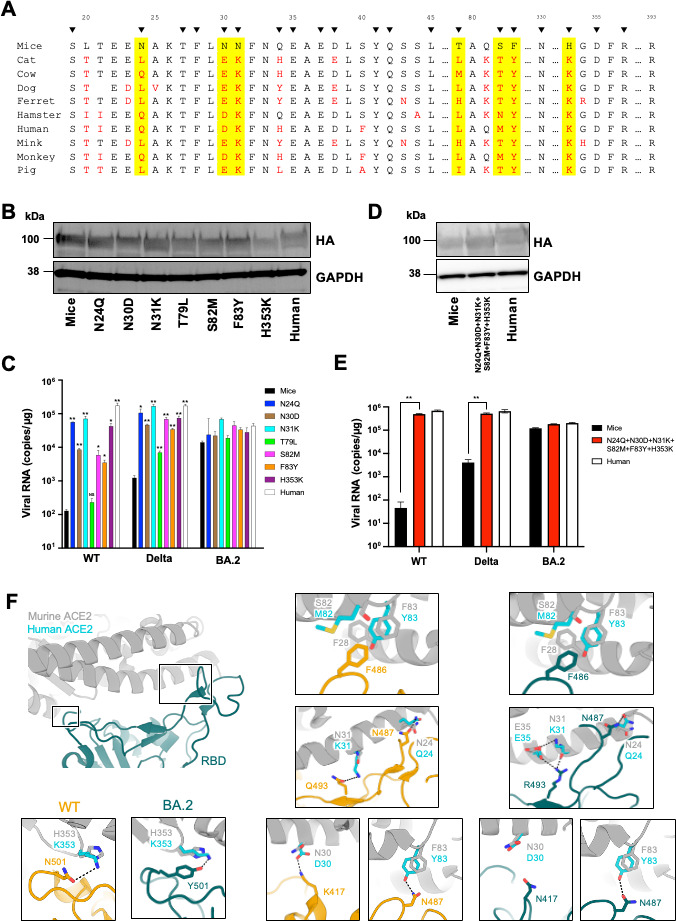
N24Q, N30D, N31K, S82M, F83Y, and H353K mutations allow murine ACE2 to support SARS-CoV-2 infection. (**A**) Comparison of the ACE2 amino acid residues that interface with the RBD of SARS-CoV-2. Amino acid differences from murine ACE2 are represented in red, and amino acid substitutions unique to murine ACE2 are highlighted in yellow. Black arrows indicate the residues in ACE2 that interact with SARS-CoV-2 spike as previously reported (17). The alignment was performed by T-COFFEE (35). (**B**) The expression of the murine HA-tagged ACE2 mutants in HEK293T cells was confirmed by western blot using an anti-HA antibody. (**C**) Intracellular viral RNA was assessed in cells expressing individual murine ACE2 mutants (N24Q, N30D, N31K, T79L, S82M, F83Y, or H353K) following inoculation with SARS-CoV-2 carrying the WT, Delta, or BA.2 spike protein. Experiments were done in triplicate. ^*^P < 0.05 and ^**^P < 0.01. (**D**) The expression of HA-tagged human, murine, or mutant murine ACE2 in HEK293T cells was confirmed by western blot using an anti-HA antibody. (**E**) Intracellular viral RNA was assessed in cells expressing mutant murine ACE2 with six mutations (N24Q, N30D, N31K, S82M, F83Y, and H353) following inoculation with SARS-CoV-2 carrying the WT, Delta, or BA.2 spike protein. Experiments were done in triplicate. ^**^P < 0.01. (**F**) Interactions between residues 24, 30, 31, 82, 83, and 353 of ACE2 with the SARS-CoV-2 spike RBD. Structures of ACE2 (gray), BA.2 spike (dark green), and WT spike (orange) are shown in the large ribbon model panel. In the insets, the amino acid residues at positions 24, 28, 30, 31, 35, 82, 83, and 353 in ACE2 are depicted in cyan for human and gray for mouse ACE2. These residues, along with those of their interaction partners in the SARS-CoV-2 spike RBD, are shown as stick models. Structures of WT RBD-hACE2 (PDB ID; 6LZG), BA.2 RBD-hACE2 (PDB ID; 8DM6), and BA.2 RBD-mACE2 (PDB ID; 7XOC) were obtained from the Protein Data Bank (PDB). For the structure of WT RBD-mACE2, a predictive model of the interaction was constructed by superimposing hACE2 and mACE2 (PDB ID; 7XOC) in the structure of WT RBD-hACE2.

To identify the amino acid determinants in murine ACE2 that affect its ability to support SARS-CoV-2 entry, we aligned the amino acid sequence of the regions in the 10 mammalian ACE2 orthologs used in our *in vitro* experiments that interact with SARS-CoV-2 spike (Fig. 4A) (32–34). Among 20 amino acid residues directly interacting with the spike protein, we identified seven unique residues (N24, N30, N31, T79, S82, F83, and H353) in murine ACE2 (highlighted in yellow).

To analyze the effects of these residues on SARS-CoV-2 infectivity, we generated seven expression plasmids, each encoding a mutated murine ACE2 where one of the seven residues had been changed to match the human sequence (N24Q, N30D, N31K, T79L, S82M, F83Y, and H353K). After confirming the expression of these mutated ACE2s in cells by western blotting (Fig. 4B), we assessed infection using WT and Delta SARS-CoV-2, which our earlier experiments demonstrated murine ACE2 does not support, and BA.2, which we showed murine ACE2 can support. We found that cells expressing the N24Q, N30D, N31K, S82M, F83Y, or H353K mutants were more susceptible to WT and Delta SARS-CoV-2 than those expressing WT murine ACE2. However, for BA.2 infection, no significant difference was observed between the mutant and WT murine ACE2 (Fig. 4C). To further evaluate the effects of these mutations, we made a murine ACE2 containing all six of the mutations that resulted in increased susceptibility (N24Q, N30D, N31K, S82M, F83Y, and H353K) and confirmed its expression by western blot (Fig. 4D). Then, we infected cells expressing this mutant ACE2 with WT, Delta, or BA.2 SARS-CoV-2, which has caused pandemics worldwide in the past. Notably, the cells expressing the mutant murine ACE2 with six mutations had similar susceptibility as those expressing human ACE2 (Fig. 4E).

Next, we used structural information to explore how mutating these six residues in murine ACE2 might affect interactions with the SARS-CoV-2 RBD. Residue N487 in the SARS-CoV-2 BA.2 and WT spike RBDs can interact with human ACE2 Q24 (34, 36). Thus, the murine ACE2 N24Q mutant would possibly also have improved binding affinity to BA.2 and WT spike (Fig. 4F).

K417 in the WT spike RBD can form a salt bridge with D30 in human ACE2 (34), but our modeling suggested that this interaction would not occur with the N30 in murine ACE2 (Fig. 4F) (37). In contrast, N417 in the BA.2 spike RBD does not interact with D30 in human ACE2 and N30 in murine ACE2 (Fig. 4F) (34, 37). Consequently, the murine ACE2 N30D mutation would likely enhance binding affinity with the WT but not the BA.2 spike protein.

As Q493 in the WT spike RBD is expected to form a hydrogen bond with K31 in human ACE2 (34), no interaction would be expected with the N31 present in murine ACE2 (Fig. 4F). Therefore, the N31K mutation in murine ACE2 would likely contribute to increased binding affinity with WT spike. Residue 493 in the BA.2 spike protein is Arg, which forms a hydrogen bond with N31 in murine ACE2 (37). However, the K31 in human ACE2 does not have this interaction with BA.2 R493. Instead, two salt bridges form with BA.2 R493 via human ACE2 residues K31 and E35 (36). As a result, the interaction between murine ACE2 and BA.2 spike would be maintained whether the residue at position 31 was mutated to Lys or not (Fig. 4F).

The residues F28, L79, M82, and Y83 in human ACE2 form the hydrophobic interaction with F486 in WT Spike RBD (34). S82M in murine ACE2 would enhance this hydrophobic interaction, and F83Y in murine ACE2 would additionally form a hydrogen bond with N487 in SARS-CoV-2 spike RBD (Fig. 4F). These amino acid substitutions in murine ACE2 likely contributed to the susceptibility to SARS-CoV-2 WT spike by increasing the number of interactions.

Residue N501 in the WT spike RBD is expected to interact with K353 in human ACE2 (38) but not with the H353 in murine ACE2 (Fig. 4F) (37). In contrast, Y501 in the BA.2 spike RBD forms an interaction with residue 353 in ACE2 whether it is His (as in murine ACE2) or Lys (as in human ACE2; Fig. 4F) (36, 37). Accordingly, no difference in the binding affinity between murine ACE2 and BA.2 spike is expected to occur with the mutation at residue 353. Thus, the functional effects on susceptibility to WT vs BA.2 SARS-CoV-2 that we observed for the six amino acid substitutions—N24Q, N30D, N31K, S82M, F83Y, and H353K—align with the expected consequences that these mutations would have on the structural interaction between ACE2 and the viral RBD.

### Cells expressing ACE2 from a species closely related to mice are susceptible to SARS-CoV-2 variants that cannot utilize murine ACE2

**Fig 5:**
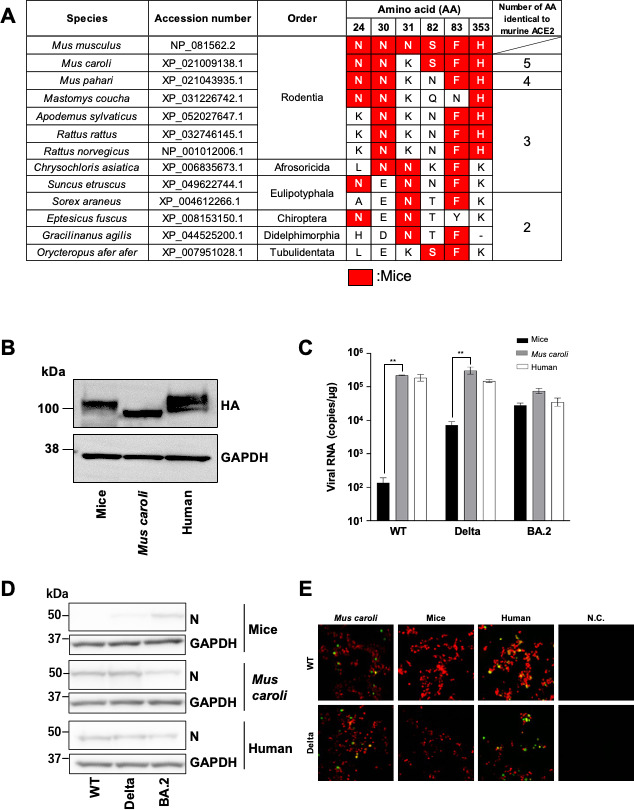
The susceptibility of cells expressing ACE2 from a closely related species of mice to SARS-CoV-2. (**A**) Species with two or more residues that match those at positions 24, 30, 31, 82, 83, and 353 in murine ACE2. (**B**) Expression of HA-tagged murine, *Mus caroli*, and human ACE2 was confirmed by western blot. (**C**) Intracellular viral RNA was assessed in cells expressing *Mus caroli* ACE2 after inoculation with SARS-CoV-2 carrying the spike protein of WT, Delta, or BA.2. Experiments were done in triplicate. ^*^P < 0.05 and ^**^P < 0.01. (**D and E**) Murine, *Mus caroli*, or human ACE2-expressing HEK293T cells were infected with SARS-CoV-2 carrying the spike protein of WT, Delta, and BA.2. At 24 h.p.i., infection was assessed by western blot of viral nucleocapsid protein (**D**) and immunofluorescence of GFP from the recombinant chimeric SARS-CoV-2 (E; HA-tagged ACE2 in red and GFP signal in green).

To investigate species with residues identical to those of murine ACE2 at positions 24, 30, 31, 82, 83, and 353, we conducted an amino acid alignment of 185 mammalian ACE2 orthologs (Table S1). We found that six species had N at position 24 in ACE2; 10 had N at position 30; 24 had N at position 31; eight had S at position 82; 30 had F at position 83; and nine had H at position 353. In four species—including those in the orders Eulipotyphala (*Sorex araneus*), Chiroptera (*Eptesicus fuscus*), Didelphimorphia (*Gracilinanus agilis*), and Tubulidentata (*Orycteropus afer afer*)— the ACE2 sequences aligned with murine ACE2 sequence at two of the six residues (Fig. 5A). Six species, four belonging to Rodentia (*Mastomys coucha*, *Apodemus sylvaticus*, *Rattus rattus*, and *Rattus norvegicus*), one belonging to Afrosoricida (*Chrysochloris asiatica*), and one belonging to Eulipotyphala (*Suncus etruscus*), had three of the six residues of interest match those seen in murine ACE2. Only one species each belonging to Rodentia (*Mus pahari* and *Mus caroli*) had four or five of six residues that match those of the murine ACE2 sequence. No species among those we examined matched murine ACE2 at all six positions of interest.

To continue our investigation of SARS-CoV-2 host tropism, we chose ACE2 from *Mus caroli* for further analysis since the overall amino acid sequence was most similar to that of mouse among the orthologs we examined (97.52%). As before, we cloned *Mus caroli* ACE2 and confirmed expression by western blot (Fig. 5B) and then assessed its ability to support entry of WT, Delta, and BA.2 SARS-CoV-2. Intracellular viral RNA levels after WT and Delta infection were significantly higher in cells expressing *Mus caroli* ACE2 vs murine ACE2 (Fig. 5C). Indeed, viral RNA levels with *Mus caroli* ACE2 were comparable to those observed with human ACE2. Viral RNA levels with BA.2 infection were comparably high regardless of the ACE2 ortholog used.

Nucleocapsid protein was also strongly detected by western blot in cells expressing *Mus caroli* ACE2 for all three spike protein variants assessed (Fig. 5D). Immunofluorescence analysis provided further evidence of WT and Delta infection occurring in cells expressing *Mus caroli* ACE2 (Fig. 5E). Together, these results suggest that cells expressing *Mus caroli* ACE2 are susceptible to SARS-CoV-2 variants that are not supported by murine ACE2.

## DISCUSSION

Interaction between the spike protein of SARS-CoV-2 and the host receptor ACE2 is a key determinant of host tropism as it dictates whether viral entry will successfully occur. Since the onset of the pandemic, numerous variants of SARS-CoV-2 have emerged. In the present study, we found that, with the exception of mouse, ACE2 orthologs from multiple species that humans are commonly in contact with can support the entry of SARS-CoV-2 expressing the spike protein from 10 different VOCs/VOIs. These data suggest that there might be broad species susceptibility to SARS-CoV-2 variants (Fig. 1C through K). Previous studies have already documented natural infections of the WT, Alpha, and Delta variants in white-tailed deer, as well as the Delta variant in hamsters (13, 14, 39, 40). Moreover, transmission of SARS-CoV-2 from minks and hamsters to humans has been reported (12, 14, 41). Having additional reservoirs of the virus may lead to the emergence of new variants with the potential for increased spread among humans and the start of a new COVID wave.

Unlike the other orthologs in our study which supported infection of all the 10 variants examined, cells expressing murine ACE2 were not susceptible to WT, Delta, or Lambda SARS-CoV-2 (Fig. 1H, L and M). These findings are consistent with previous mouse studies that demonstrated a lack of infection by WT SARS-CoV-2 or the Delta variant *in vivo* (26).

Previous studies have also shown that mice are susceptible to infection with Alpha and Beta SARS-CoV-2 variants, indicating a role for the N501Y mutation that is found in the spike protein of these variants in determining infectivity in mice (18–20, 26). Our analysis supports this hypothesis, as all variants capable of utilizing murine ACE2 for host cell entry shared the N501Y mutation in the spike protein (Fig. 2A). In this study, we showed that inserting the N501Y mutation in the WT spike protein still allowed for viral entry, while reversing this mutation in Alpha back to the WT residue resulted in a loss of susceptibility in murine ACE2-expressing cells (Fig. 2C through E) and a loss of infectivity in mice *in vivo* (Fig. 3C). These data suggest that the N501Y mutation in the spike protein is essential for the utilization of murine ACE2 by the Alpha variant.

We also assessed the N501Y mutation in the context of the murine-adapted SARS-CoV-2 MA10, which was generated after 10 passages in BALB/c mice inoculated with SARS-CoV-2 MA (31). The MA10 spike protein contains the mutations Q493K, Q498Y, and P499T, and we are not aware of studies that compared in mice the infectivity of SARS-CoV-2 containing the MA10 spike protein vs the N501Y mutation. Our experiments suggest that the N501Y mutation has a comparable effect on infectivity in mice as the three mutations in the MA10 spike protein (Fig. 3E).

Our comparison of amino acid residues in mammalian ACE2 orthologs identified seven candidate residues in murine ACE2 that might restrict the entry of the WT, Delta, and Lambda variants (Fig. 4A) (23, 32–34). Based on our experimental data, we found six residues in murine ACE2 that, when altered to the amino acid found in human ACE2 (N24Q, N30D, N31K, S82M, F83Y, and H353K), enabled entry of the WT and Delta variants (Fig. 4C). A previous study demonstrated that ACE2 with N30D, N31K, F83Y, and H353K mutations in murine ACE2 enhances susceptibility to WT SARS-CoV-2 (21, 22, 24, 25). At this time, we newly identified that N24Q and S82M in murine ACE2 significantly enhance susceptibility to SARS-CoV-2.

The six residues identified in this study are located at the interface between ACE2 and the spike protein. To understand the possible mechanism by which these six amino acid substitutions allow murine ACE2 to facilitate SARS-CoV-2 entry, we used available structural information to determine how these changes would affect interaction with the viral RBD. Our analyses predicted the formation of novel interactions between WT spike and murine ACE2 carrying either N24Q, N30D, N31K, S82M, F83Y, or H353K (Fig. 4H). This is consistent with the report of Rawle et al. (21) and likely contributes to the inability of SARS-CoV-2 WT to infect mice. These results suggest that these six murine ACE2 amino acids are important determinants of SARS-CoV-2 WT infection in mice.

Based on our findings, we hypothesized that these six amino acids in murine ACE2 play a crucial role in the host range of SARS-CoV-2. Thus, we hypothesized that animals possessing these same six amino acid residues may also exhibit different susceptibility to SARS-CoV-2 variants. We compared positions 24, 30, 31, 82, 83, and 353 in mammalian ACE2 orthologs and found eight species that shared three or more of these amino acids with murine ACE2 (Fig. 5A). Six of these species belong to Rodentia, the same order as mice (Fig. 5A). Rodentia are considered a major animal reservoir of coronaviruses, including SARS-CoV-2 (42). A previous study using phylogenetic clustering and sequence alignment suggested that ACE2 orthologs of Rodentia species, including that of *Mus caroli*, would not mediate SARS-CoV-2 infection (43). However, interestingly, in our experiments, HEK293T cells expressing *Mus caroli* ACE2 were susceptible to recombinant SARS-CoV-2 carrying the WT or Delta spike protein, both of which could not infect mice (Fig. 5C through E). Considering that *Mus caroli* is the species most closely related to mice, there is a possibility that among Rodentia, only mice (*Mus musculus*) exhibit different susceptibility to SARS-CoV-2 variants.

Numerous SARS-CoV-2 variants have emerged since the onset of the pandemic. The Omicron variant harbors an exceptionally high number of mutations, many of which were rarely observed in previous SARS-CoV-2 variants, leading to hypotheses about its evolutionary history (44, 45). One such hypothesis suggests that Omicron may have accumulated mutations in an animal host before spilling back into humans (15, 16). Our study indicates that the insusceptibility of mice to SARS-CoV-2 is a highly unusual phenomenon, and a wide range of species, including rodents, could serve as potential reservoirs for SARS-CoV-2. These findings underscore the importance of monitoring the circulation and evolution of SARS-CoV-2 in rodent populations to better understand its transmission dynamics and potential zoonotic risks.

In conclusion, our study identified that the N501Y mutation in the SARS-CoV-2 spike protein allows for utilization of murine ACE2 and infection of mice with SARS-CoV-2 *in vivo*. Furthermore, we identified six amino acid residues (N24, N30, N31, S82, F83, and H353) in murine ACE2 that restrict viral entry. Moreover, cells expressing ACE2 from a species closely related to mice were susceptible to SARS-CoV-2 variants that do not infect mice, suggesting that only mice among Rodentia exhibit different susceptibility to SARS-CoV-2 variants. Our study provides new insight into understanding the determinants of the host range of SARS-CoV-2, contributing to efforts to prevent future transmission and infection.

## MATERIALS AND METHODS

### Plasmids

Full-length cDNA of human ACE2 and human TMPRSS2 was amplified from a cDNA library derived from HEK293 C34 cells (28). Macaque and ferret ACE2-expressing plasmids were purchased (Cat# RDC3079 and RDC3148; R&D systems, MN, USA) and modified based on the sequence acquired from GenBank (Table S2). Full-length cDNAs of cat, cow, dog, ferret, hamster, mink, macaque, mouse, pig, and *Mus caroli* ACE2 were synthesized by Thermo Fisher Scientific (MA, USA) based on the sequence deposited in GenBank (Table S2). All mutations of murine and human ACE2 were generated by using PCR with the respective primers (provided upon request). All cloned ACE2 described above were inserted at the XhoI site of the pCSII-EF-HA vector (46) by using In-Fusion︎ HD Cloning Kit (Cat# 638933; Takara Bio, Siga, Japan). The cDNA clone of human TMPRSS2 was inserted at the XhoI site of the vector pCSII-EF-Flag using In-Fusion︎ HD Cloning Kit. Primers used in this study can be provided upon request.

### Cell line

HEK293T cells were cultured at 37°C in Dulbecco's modified Eagle's medium (DMEM) (high glucose; Cat# 08488–55; Nacarai Tesque, Kyoto, Japan) supplemented with 1% penicillin-streptomycin (PS) mixed solution (Cat# 09367–34; Nacarai Tesque) and 10% fetal bovine serum (Cat# FB-1280/500; Biosera, Nuaillé, France). VeroE6/TMPRSS2 cells were maintained in DMEM (low glucose; Cat# 041–29775; FUJIFILM Wako Chemicals, Osaka, Japan) supplemented with 1 mg/mL G418 (Cat# 09380–44; Nacarai Tesque) and 10% fetal bovine serum. HEK293 C34 cells were maintained in DMEM (high glucose) supplemented with 1% PS mixed solution, 10 µg/mL Blasticidin (solution; Cat# ant-bl, InvivoGen, CA, USA), and 10% fetal bovine serum. Cells were maintained under humidified conditions and 5% CO_2_.

### SARS-CoV-2 reverse genetics

Chimeric recombinant SARS-CoV-2 carrying the superfolder GFP gene and MA10-based chimeric recombinant SARS-CoV-2 were generated by CPER as previously described (28, 47, 48). In brief, nine DNA fragments encoding the partial genome of SARS-CoV-2 B.1.1 (WT; GISAID ID: EPI_ISL_408667) were prepared by PCR using KOD One PCR Master Mix Blue (Cat#KMM-201, TOYOBO, Osaka, Japan). A linker fragment encoding hepatitis delta virus ribozyme, bovine growth hormone poly A signal, and cytomegalovirus promoter was also prepared by PCR. The 10 obtained DNA fragments were mixed and used for CPER. To generate recombinant SARS-CoV-2 MA10, mutations were inserted in fragments 3, 4, 8, and 9. Moreover, to generate the WT-based or MA10-based chimeric recombinant SARS-CoV-2, fragment eight encoding the spike protein of WT or MA10 was replaced by fragment eight encoding the spike protein of the respective SARS-CoV-2 variants. The sequences of spike protein from SARS-CoV-2 variants were acquired from GISAID (Table S3). The CPER products were transfected into HEK293 C34 cells using TransIT-LT1 (Cat# MIR2300; Takara bio) according to the manufacturer’s instructions (28). One day post-transfection, the culture medium was replaced with DMEM (high glucose) containing 2% FBS, 1% PS, and doxycycline at 1 mg/mL (Cat# 1311N; Takara Bio). Six days post-transfection, the culture medium was harvested and centrifuged (850 × *g*, 3 min), and the supernatants were collected as the seed virus. All viruses were kept at −80°C until use.

### Western blotting

HEK293T cells were seeded in a 24-well plate. After 24 h, HA-tagged ACE2 orthologs were transfected using the TransIT-LT1 reagent according to the manufacturer’s instructions. The cells were rinsed once with PBS (Cat# 14249–24; Nacarai Tesque) 24 h post-transfection and then lysed in 2 × Laemmli sample buffer (Cat# 1610737; Bio-Rad Laboratories, Hercules, CA, USA) supplemented with 5% 2-mercaptoethanol (Cat# 19–1335-2; Sigma-Aldrich, MO, USA) and incubated for 5 min at 95°C. The samples were then subjected to western blot analysis. SDS-PAGE was performed using e-PAGEL (Cat# E-T1020; ATTO CORPORATION, Tokyo, Japan) and transferred to polyvinylidene fluoride transfer membrane (Cat# IPVH00010; Millipore, MA, USA). Membranes were blocked for 1 h at room temperature with 5% skim milk (Cat# 190–12865; Wako, Osaka, Japan) and then immunoblotted with specific antibodies (see below) and subsequently incubated with horseradish peroxidase-conjugated antibody against mouse immunoglobulin (Cat# 115–0035-003; Jackson ImmunoResearch Laboratories, PA, USA), followed by detection with enhanced chemiluminescence (ECL) substrate (Cat# 34095; Thermo Fisher Scientific). The following antibodies were used in this study: anti-GAPDH (Cat# NBP2-27103H; Wako), anti-HA (Cat# 901502; BioLegend, CA, USA), anti-Flag (Cat# F3165; Sigma-Aldrich), and SARS-CoV-2 nucleocapsid antibody (Cat# 40413-MM05; Sino Biological, Kanagawa, Japan). All antibodies are diluted in PBS-T with 5% skim milk.

### SARS-CoV-2 preparation and titration

The working virus stocks of chimeric recombinant SARS-CoV-2 were prepared and titrated as previously described (28). In brief, all viruses were amplified in VeroE6/TMPRSS2 cells, and the culture supernatants were harvested and stored at −80°C until use. The infectious titers of the culture supernatants were determined by TCID_50_ (49). Nucleotide sequences were confirmed by SeqStudio Genetic Analyzer (Thermo Fisher Scientific) or outsourced service (Fasmac, Kanagawa, Japan). The sequence data were analyzed by SnapGene v7.0 software (GSL Biotech LLC, CA, USA).

### SARS-CoV-2 infection

HEK293T cells were seeded in a 24-well plate. After 24 h, ACE2 orthologs and human TMPRSS2 at 0.25 µg each with 1 µL of TransIT-LT1 reagent were used for transfection. After 24 h, the culture medium was replaced with DMEM (2% FBS and 1% PS), and the cells were inoculated with SARS-CoV-2 (MOI = 0.5 or 0.1 or 0.01) and incubated at 37°C for 1 h. Cells were washed once with DMEM (2% FBS and 1% PS), and fresh medium was then added (DMEM, 2% FBS and 1% PS). After incubating for 12 or 24 h, the infected cells were harvested and subjected to RT-qPCR, western blotting, or immunofluorescent microscopy.

### Immunofluorescent microscopy

Infected cells were fixed with 4% paraformaldehyde phosphate buffer solution (Cat# 09154–85; Nacalai Tesque), permeabilized with 0.25% saponin (Cat# 30502–42; Nacalai Tesque), and then incubated with anti-HA tag antibody (Cat# 901502; BioLegend, CA, USA) and anti-GFP antibody (Cat# 598; Medical and Biological Laboratories, Tokyo, Japan) followed by the secondary antibodies Alexa Fluor Plus 594 (Cat# A48288; Thermo Fisher Scientific) and Alexa Fluor Plus 488 (Cat# A48282; Thermo Fisher Scientific). Coverslips were mounted onto glass slides with Vectashield (Cat# H-1000–10; Vector Laboratories, CA, USA) and sealed with nail polish. The image was taken using BZ-X810 (KEYENCE, Osaka, Japan).

### RT-qPCR

For the quantification of viral RNA copies, total RNA was extracted from cells using a PureLink RNA Mini Kit (Cat# 12183018A; Thermo Fisher Scientific), and then first-strand cDNA synthesis and RT-qPCR were performed with One Step PrimeScript RT-qPCR Kit (Cat# RR064B; Thermo Fisher Scientific) according to the manufacturer’s instructions. Fluorescent signals were acquired using a QuantStudio 3 Real-Time PCR system (Thermo Fisher Scientific).

### Animal experiments

BALB/cAJcl mice (male, 8 weeks old) were purchased from Japan CLEA Inc. (Tokyo, Japan). Syrian hamsters (male, 4 weeks old) were purchased from Japan SLC Inc. (Shizuoka, Japan). For the viral infection experiments, mice and hamsters were anesthetized by intraperitoneal injection of a mixture of 0.15 mg/kg medetomidine hydrochloride (Domitor, Nippon Zenyaku Kogyo), 2.0 mg/kg alphaxalone (Alfaxan, Jurox), and 2.5 mg/kg butorphanol (Vetorphale, Meiji Seika Pharma). The chimeric recombinant SARS-CoV-2 (WT, WT-N501Y, Alpha, Alpha-Y501N, MA10, MA10-WT, and MA10-WT-N501Y) was intranasally inoculated (5,000 TCID_50_ in 50 µL for mice; 10,000 TCID_50_ in 100 µL for hamsters) under anesthesia. Lungs were anatomically collected at 3 d.p.i. and homogenized by BioMasher2 (Cat# 320103; Nippi, Tokyo, Japan). Viral RNA was extracted by PureLink RNA Mini Kit (Cat# 12183018A; Thermo Fisher Scientific) and subjected to RT-qPCR.

### Sequence analysis

Multiple alignment of amino acid sequence of ACE2 orthologs or spike gene of SARS-CoV-2 variants was performed using T-COFFEE (35) or the Constraint-based Multiple Alignment Tool (COBALT) (50).

### Statistical analysis

Results are expressed as mean ± SDs. The significance of differences in the means was determined by the Wilcoxon rank sum test using GraphPad Prism 9 (GraphPad Software, MA, USA).

## Data Availability

The virus strains and ACE2 sequences used in this study are registered in GenBank and GISAID. Each accession number is listed in the supplementary material (Tables S1 to S3).

## SUPPLEMENTAL MATERIAL

Table S1 jvi.00543-25-s0001.xlsx Amino acid sequences of mammalian ACE2 from GenBank.

Table S2 jvi.00543-25-s0002.xlsx Nucleotide sequences of animal ACE2 constructed in this study.

Table S3 jvi.00543-25-s0003.xlsx Nucleotide sequences of VOC/VOIs from GISAID.

ASM does not own the copyrights to Supplemental Material that may be linked to, or accessed through, an article. The authors have granted ASM a non-exclusive, world-wide license to publish the Supplemental Material files. Please contact the corresponding author directly for reuse.
